# Flexible Insole Sensors with Stably Connected Electrodes for Gait Phase Detection

**DOI:** 10.3390/s19235197

**Published:** 2019-11-27

**Authors:** Wenzheng Heng, Gaoyang Pang, Feihong Xu, Xiaoyan Huang, Zhibo Pang, Geng Yang

**Affiliations:** 1 State Key Laboratory of Fluid Power and Mechatronic Systems, School of Mechanical Engineering, Zhejiang University, Hangzhou 310027, China; wenzhengheng@zju.edu.cn (W.H.); gaoyangpang@zju.edu.cn (G.P.); 2 Institute of Mechanical Manufacturing Technology, China Academy of Engineering Physics, Mianyang 621900, China; xufeihong18@gscaep.ac.cn; 3 College of Electrical Engineering, Zhejiang University, Hangzhou 310027, China; xiaoyanhuang@zju.edu.cn; 4 ABB Corporate Research Sweden, 72178 Vasteras, Sweden; pang.zhibo@se.abb.com

**Keywords:** gait phase, insole sensors, surface processing, inkjet printing, visual-based reference, machine learning

## Abstract

Gait analysis is an important assessment tool for analyzing vital signals collected from individuals and for providing physical information of the human body, and it is emerging in a diverse range of application scenarios, such as disease diagnosis, fall prevention, rehabilitation, and human–robot interaction. Herein, a kind of surface processed conductive rubber was designed and investigated to develop a pressure-sensitive insole to monitor planar pressure in a real-time manner. Due to a novel surface processing method, the pressure sensor was characterized by stable contact resistance, simple manufacturing, and high mechanical durability. In the experiments, it was demonstrated that the developed pressure sensors were easily assembled with the inkjet-printed electrodes and a flexible substrate as a pressure-sensitive insole while maintaining good sensing performance. Moreover, resistive signals were wirelessly transmitted to computers in real time. By analyzing sampled resistive data combined with the gait information monitored by a visual-based reference system based on machine learning method (k-Nearest Neighbor algorithm), the corresponding relationship between plantar pressure distribution and lower limb joint angles was obtained. Finally, the experimental validation of the ability to accurately divide gait into several phases was conducted, illustrating the potential application of the developed device in healthcare and robotics.

## 1. Introduction

Gait monitoring is able to diagnosis certain diseases by analyzing human gait patterns, especially for chronic diseases threatening the elderly in the aging society [1,2]. Fall prevention for disabled and older people is another essential application of gait monitoring [3]. Moreover, gait monitoring plays an important role in athletic training to help athletes improve their performance [4,5] and in the gait design and control of bipedal humanoid robots [6,7]. In addition, gait phase is broadly investigated in the field of wearable exoskeleton robots [8,9], because only when the rotation movement of the exoskeleton’s joints changes reasonably with the human gait can it provide favorable walking assistance and comfort for the wearer [10].

In the versatile application scope, many significant efforts have been devoted to gait detection. The imaging processing method is one of the effective ways to capture the visual information of gait cycles [11]. However, it has spatial and temporal restrictions that limit the analysis to be at a specific time and/or in a confined location [12]. To estimate temporal gait parameters, Lee et al. developed a compactable wireless system based on two three-axis inertial sensors [13]. Although inertial sensors are lightweight and can be conveniently mounted on the human body, compared with image-based methods, there are some limitations with the quantity and the accuracy of motion signals captured by inertial sensors [14]. The electromyography (EMG) sensors are used to classify a person’s gait by collecting electromechanical signals directly reflecting the user’s motion intentions [15]. However, the signals are unstable and time-varying due to muscle fatigue and electrodes degradation [16]. Recently, wireless wearable devices have been developed rapidly in motion capture and health monitoring [17,18]. Insole pressure sensors, as a kind of wearable sensor, have shown great potential for future applications [3]. They are convenient, portable, unobtrusive, and versatile, providing a rich source of information for real-time gait analysis in both indoor and outdoor environments [19]. For example, by leveraging a smart insole system, Najafi et al. [20] exploited a comprehensive investigation of adherence to alert-based cues of plantar pressure offloading in patients with diabetic foot disease during activities of daily living. However, the effectiveness of the insole system was validated by setting risk pressure thresholds rather than by providing sufficient gait information [20].

Here, we proposed an effective and low-cost laser processing method to modify the surface of conductive rubber, enhancing the stability of sensing performance when sensing units contact with electrodes directly. The developed conductive rubber was further integrated as an insole device that was experimentally validated by sampling planar pressure in real time. The accuracy of the divided gait phase was verified adequately by the image-based method. The primary novelties of this work are listed as follows: (1) a novel method for stabilizing surface resistance of conductive rubber through the laser surface modification method; (2) a detailed feature and fabrication process of sensor units, characterization of sensor units, and experimental validation of the developed flexible insole sensor; and (3) integrating plantar pressure information and visual information for analysis and comparison.

This paper is organized as follows. Section 2 presents the related state-of-the-art work, including wearable insole sensors and light algorithms. Section 3 describes the structure and fabrication process of the flexible pressure sensing unit. Section 4 discusses the experimental results, including resistance before and after surface processing, electrical characteristics of sensing units and inkjet-printed electrodes, data acquisition and data analysis of a pressure-sensitive insole, construction of a visual reference system, and the validation of flexible insole sensors. Section 5 evaluates and analyzes the proposed framework. Finally, the future work (Section 6) and a brief conclusion (Section 7) establish that the vision aided intelligent insole platform will later collect more data of the planar pressure and the joint angles of the lower body for gait analysis to find the connection between the two parameters by machine learning.

## 2. Related Work

Nowadays, most available smart insoles are embedded with force sensitive sensors (FSRs) that are ultra-thin, flexible, and have good linearity. Nevertheless, the FSR also has its limitations. Firstly, FSRs are not cost-effective [21]. Secondly, FSRs are prone to breakage via fatigue stress because the electrodes directly bear alternating load induced by the external force applied to the ultra-thin sensor [10]. Thirdly, the drift and hysteresis error of FSRs is ten to one hundred times higher than that of the load cell [22]. Another promising method is to embed insoles with six-axis force sensors. For instance, Ishiguro et al. [23] presented a control system where the force sensors were embedded into two insoles to construct an interface for a human teleoperator to control a humanoid robot leg. However, most commercial six-axis force sensors are expensive, and they are easily broken by loading with long-term and heavy force because they are made of rigid components, especially when they are used for gait monitoring [24]. With the development of functional materials, many materials show great potential for pressure sensing [25]. Piezoresistive conductive rubber is a kind of promising flexible material to be complementary to FSRs since, during the fabrication process, it can be reinforced to reduce creep by adding carbon nanotubes [26] or by changing the electrode configuration of the integrated sensor [27]. Some researchers have demonstrated that conductive rubber has a long lifetime [28] and low production cost [29]. Generally, the traditional conductive rubber sensors are usually connected with electrodes by using silver conductive paste or polymer adhesive, which can effectively reduce the impact of contact resistance. However, this connection mode easily falls off due to fatigue, especially when it is used for an insole sensor for long-term and high-load monitoring. Saito et al. [30] used conductive rubber to make a pressure-sensitive insole directly in contact with electrodes, but the resistance data fluctuated during static calibration.

In terms of lightweight algorithms of machine learning methods for wearable applications, Krause et al. [31] deployed machine leaning methods based on Kohonen Self-Organizing Maps (KSOMs) and k-Means clustering on a mobile phone to realize a classifier to identify user states and to automatically modify algorithms’ settings based on experience. LeMoyne et al. [32] utilized the multilayer perceptron neural network as a machine learning platform to classify gait patterns based on data from wearable inertial sensors. However, the data of the above methods were post-processed in additional computer or cloud computing resources. To reduce the network and privacy cost of sending data to the cloud and to make machine-learning algorithms scalable, Rawassizadeh et al. [33] presented lightweight algorithms to identify daily human behavioral patterns on small wearable devices. All these efforts illustrate the feasibility to leverage machine learning methods to recognize physical activity based on wearable devices. In this work, machine-learning classification, as a popular method, was used to validate the feasibility of developed flexible insole sensors for gait phase detection.

## 3. Materials and Methods

### 3.1. Structure Design

**Figure 1:**
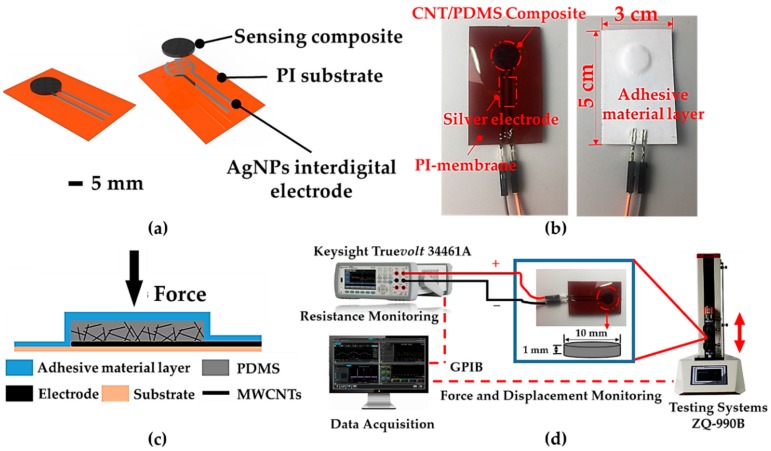
The structure design of a sensing unit and the experimental setup. (**a**) Schematic illustration of a sensing unit (scalar bar: 5 mm). (**b**) Photograph of a sensing unit after being packaged. (**c**) The layout of a pressure-sensitive unit. (**d**) A performance test system for the flexible pressure sensor.

In this paper, we developed a kind of flexible pressure sensor for planar pressure detection by using multi-wall carbon nanotubes (MWCNTs) and Polydimethylsiloxane (PDMS). MWCNTs were chosen due to their electrical conductivity [34]. PDMS was chosen because it has good flexibility and the stiffness of PDMS can be adjusted by changing the hardening agent to PDMS base ratio [35], which is suitable for personalized products of users of different weights. An interdigital electrodes array was printed on the polyimide (PI) substrate, as shown in Figure 1a,b. Figure 1c presents the multi-layers of one sensing unit. From top to bottom, they are adhesive material layer (to fix the sensing unit on the flexible circuit), MWCNTs/PDMS pressure-sensitive layer (where the resistance is sensitive to applied pressure), interdigital electrodes [which are made of silver nanoparticles (AgNPs) printed by an inkjet printer], and PI substrate (as the carrier of flexible circuits and sensitive units). Here, we did not use any conductive adhesive material (such as conductive silver adhesive and conductive polymer) [36,37] but directly mounted the sensing unit on the surface of the electrode through the adhesive material layer (tape) and realized electrical connection through direct contact. The sensing mechanism can be described in the following statements. When a load pressure is applied on the top surface of the sensor, the PDMS layer deforms, and MWCNTs uniformly dispersed inside PDMS gradually close to each other. Then, the number of connected conductive paths between carbon nanotubes increase, and thus the resistance of the sensing unit declines. The sensor thus functions as a force-to-resistance transducer. The cylindrical sensing unit has a diameter of 1 cm and a height of 1 mm. As shown in Figure 1d, the resistance was detected by using a digital multimeter (DMM, Keysight Truevolt 34461A, Keysight Technologies, Shanghai, China), and the external force was applied by a testing system (ZQ-990B, Zhiqu Precision Instrument Co., Ltd., Dongguan, China). The comparison between the proposed design and the state-of-the-art design is summarized in Table 1.

### 3.2. Fabrication

**Figure 2:**
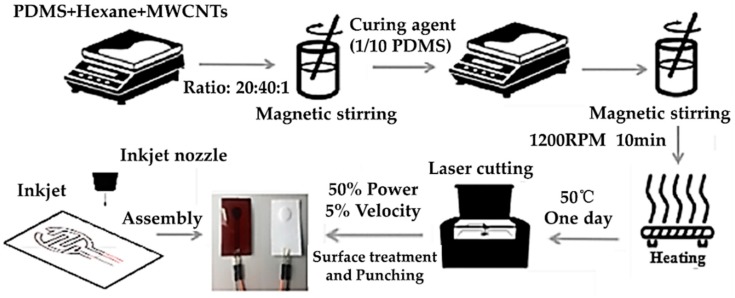
Schematic illustration of the fabrication steps of pressure sensors based on multi-wall carbon nanotubes (MWCNTs)/Polydimethylsiloxane (PDMS).

The fabrication process of the pressure-sensitive layer is shown in Figure 2. In order to obtain a sensitive conductive layer, firstly, we mixed MWCNTs (MK1858, Nanjing Muke Nano Technology Co., Ltd., Nanjing, China), PDMS prepolymer (Sylgard 184, Wuxi Changxu Technology Co., Ltd., Wuxi, China), and N-Hexane (Aladdin reagent Shanghai Co., Ltd., Shanghai, China) at a mass ratio of 20:40:1. Then, the mixed solution was stirred at 1200 RPM for 10 min to obtain a uniform mixture by using magnetic stirrer (98-1, Shanghai Sile Instrument Co., Ltd., Shanghai, China). After evenly dispersing MWCNTs in the mixture, we added a 10 wt% curing agent (in PDMS ratio) followed by magnetic stirring for 10 min. Secondly, to cure the mixture, it was placed in a vacuum oven (Bangxi Instruments Technology Co., Ltd., Shanghai, China) and heated at 50 °C for 24 h. After the curing process, we used the laser cutting machine (FD–300, Shanghai Fengying Computer Technology Development Co., Ltd., Shanghai, China) to modify the surface of the mixture at 50% of energy (in the total energy of 30 watts) and 5% of cutting speed. Finally, the shapes of pressure-sensitive units could be cut by punchers or laser cutting machine. When packaging the sensing unit, the inkjet printer (Functional nanomaterial deposition system, Shanghai Ruidu Photoelectric Technology Co., Ltd., Shanghai, China) was used to print interdigital electrodes on a PI substrate, and the number of repeated printing cycles was set to three times. Then, the sensing unit was directly placed above the inkjet-printed interdigital electrodes, the up layer of which was packaged by tape.

## 4. Results and Discussion

### 4.1. Sensing Unit Stabilizing

**Figure 3:**
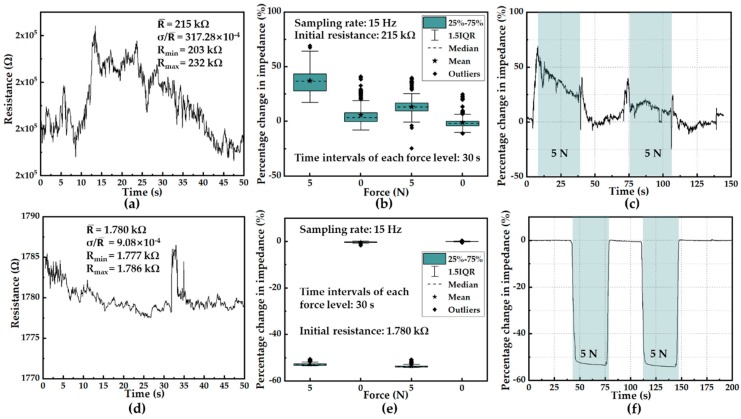
Comparison of stabilization of sensor units. (**a**) The resistance of non-processed unit without loading. (**b**) Statistical analysis of the resistance of non-processed unit loaded with two levels of force (0 N and 5 N). (**c**) The resistive response of non-processed unit loaded with two levels of force (0 N and 5 N). (**d**) The resistance of laser-processed unit without loading. (**e**) Statistical analysis of the resistance of laser-processed unit loaded with two levels of force (0 N and 5 N). (**f**) The resistive response of laser-processed unit loaded with two levels of force (0 N and 5 N).

In the fabrication process, the surface of the sensing unit was modified by a laser processing method. Before laser processing, the surface of the sensing unit was condensed with a large amount of PDMS due to gravity during the curing period. During the laser treatment, the PDMS on the surface of the sensing unit was ablated, and the surface of the sensor was covered with MWCNTs so that the sensing unit resistance significantly dropped and became stable. The resistance was detected by using the interdigital electrodes array. The test results are shown in Figure 3, in which the sample was loaded with a cyclic force ranged from 0 N to 5 N with a time interval of 30 s. It can be seen that the surface resistance had adverse impacts on the resistance in two aspects: the mean of resistance value and the fluctuation of resistance variation. In Figure 3a,d, R_max_ represents the maximum resistance during the sampling time; R_min_ represents the minimum value; $\bar{R}$ represents the average value of the resistance; σ represents the standard deviation of the resistance data. As illustrated in Figure 3a,d, after laser processing, the resistance value of the unloaded sensing unit dramatically reduced from 215 kΩ to 1.78 kΩ, and the fluctuation of resistance decreased obviously from 13.49% to 0.51%. Statistical analysis of two kinds of sensing units when loaded with the cyclic force is illustrated in Figure 3b,e. The corresponding resistive responses of two kinds of sensing units are also illustrated in Figure 3c,f to show the effects of laser processing intuitively.

**Figure 4:**
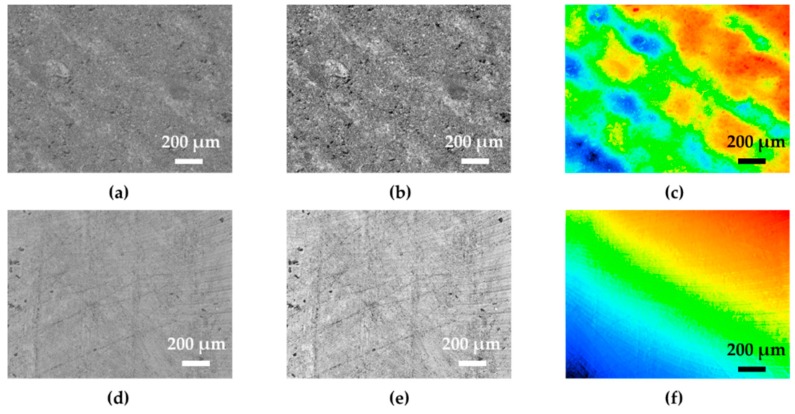
Optical images of laser-processed unit and non-processed unit. (**a**) Surface topography tracking of laser-processed unit. (**b**) Laser track of laser-processed unit. (**c**) High-low image of laser-processed unit. (**d**) Surface topography tracking of non-processed unit. (**e**) Laser track of non-processed unit. (**f**) High-low image of non-processed unit.

As shown in Figure 4, a laser confocal scanning microscope (VK-150, Keyence Corporation, Japan) was used to observe the surface topography of laser-processed and non-processed sensing units. As illustrated in Figure 4a–c, there were clear parallel traces induced by laser treatment. Compared with the results in Figure 4d–f, the surface roughness of the laser-processed unit was relatively larger than that of the non-processed unit, indicating that the polymer on the surface of the sensing unit was removed effectively, thereby enabling more MWCNTs inside PDMS to be exposed to air. Hence, when the laser-processed one directly assembled on the electrodes, it showed good stability compared to the non-processed one, since the MWCNTs of the non-processed one were mostly wrapped inside the PDMS concentrated on the surface. Therefore, it can be concluded that the static performance of the sensitive layer was effectively improved after removing the surface resistance.

### 4.2. Sensing Unit Characterization

**Figure 5:**
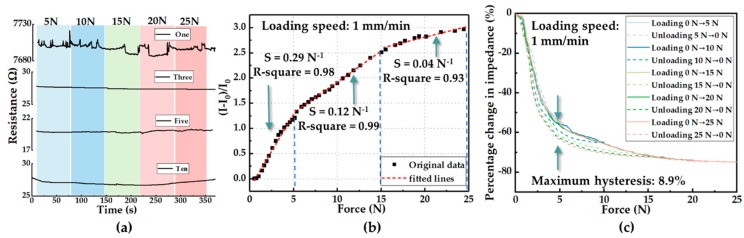
Characterization of inkjet-printed electrodes and sensing units. (**a**) The resistive response of inkjet-printed conductive traces loaded with five levels of force. (**b**) Calibration of a sensing unit (A pressure-response curve of relative change in current vs. force). (**c**) Resistive hysteresis of sensing units loaded with five kinds of force ranges.

Sensing characteristics of the sensing units were measured and analyzed under a pressure-sensing mode in both dynamic and static experiments. The resistance of the inkjet-printed electrodes decreased as the number of prints increased, as shown in Figure 5a, which was detected by a digital multimeter (DMM). At the same time, it can be seen that, under the change of external loading force (from 5 N to 25 N), the resistance of inkjet-printed electrodes—of which the printing cycle number was three or more—showed good stability. Therefore, when selecting the number of printing cycles of the interdigital electrodes, we chose to print three times to obtain stable conductive electrodes with a lower resistance.

The relative current change (defined as ΔI/I_0_, where I_0_ is the initial current value flowing through the sensing unit, and ΔI is the present current value minus the initial current value) of one sensing unit as a function of force is presented in Figure 5b. Here, we chose a force range of 0–25 N, which was equivalent to 0–320 kPa that was calculated by contact area (a circle with a diameter of 10 mm) of the sensing unit as the measure range to be consistent with daily plantar pressure. After linear fitting independently in three ranges (0–5 N, 5–15 N, and 15–25 N), the calibration result showed an approximately linear relationship between ΔI/I_0_ and applied force with three force sensitivities of S = 0.29 N^−1^, 0.12 N^−1^, and 0.04 N^−1^, respectively, where the force sensitivity is defined as S = (ΔI/I_0_)/ΔF (ΔF is the variation of force in corresponding detection range). Figure 5c shows the resistive hysteresis of the pressure sensor during five kinds of loading–unloading cycles (0–5 N, 0–10 N, 0–15 N, 0–20 N, and 0–25 N). The maximum hysteresis was approximately 8.9% at 5 N when the sensing unit was applied with a force range of 0–25 N.

**Figure 6:**
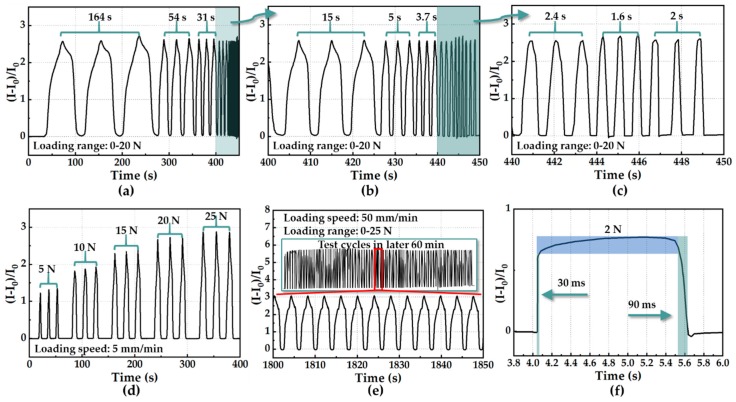
Characterization of sensing units. (**a**) Reproducibility test of the sensing unit at different time intervals. (**b**) Zoom in of results in (**a**) at the time ranged from 400 s to 450 s. (**c**) Zoom in of results in (**b**) at the time ranged from 440 s to 450 s. (**d**) Reproducibility test of the sensing unit loaded with five levels of force. (**e**) Durability test of the sensing unit loaded with a cyclic force of 0–25 N at a speed of 50 mm/min. (**f**) Transient response of the sensing unit at a force stimulus of 2 N.

**Figure 7:**
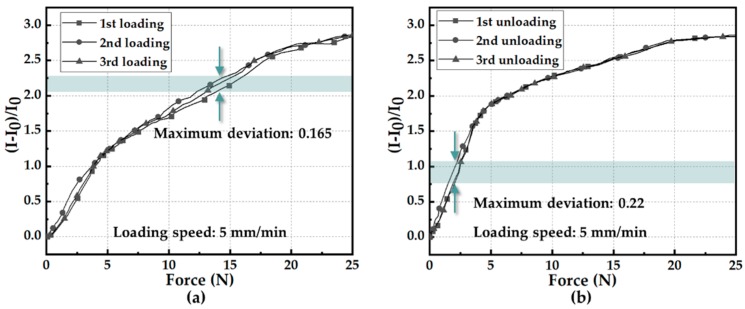
Repeatability test. (**a**) The resistive response of sensing unit from 0 to 25 N. (**b**) The resistive response of sensing unit from 25 to 0 N.

To verify the adaptability of the sensing unit to be applied to gait phase detection with different wearers and whether the frequency response of the developed sensing unit matched the normal step cadence (from 0.76–1.04 Hz in [38]; around 0.7 Hz in [40]; below 5 Hz in [41]; and around 1.18 Hz in our work, see more details in Section 5.3), an experiment of the sensing unit regarding the stability at different levels of pressure (i.e., different weights) and several time intervals (i.e., different genders) was carried out, as shown in Figure 6. Since the test machine (ZQ-990B described in Figure 1d) can only realize step inputs with a maximum loading speed of 500 mm/min, the experiment protocol here was to apply multiple levels of constant/step force with different loading speeds by leveraging the test machine. Figure 6a–c show results of the sensing response of a sensing unit at a loading range 0–20 N and nine time intervals, illustrating that the developed sensor had a frequency response range of 0.012–1.25 Hz. As illustrated in Figure 6d, the pressure sensor was capable of steadily measuring different levels of external force (5 N, 10 N, 15 N, 20 N, and 25 N). In Figure 6e, cyclic force (including loading and unloading process) between 0 and 25 N at a speed of 50 mm/min without pause was applied to the sensing unit, and loading and unloading speeds were the same. The deformation-to-time relationship could be regarded as a triangle waveform, because the load cell of test machine could only be actuated according to speed and force threshold rather than designated input (force) signal. Figure 6e shows the sensing response of the sensing unit in part of the durability test under a force range of 0–25 N (or a pressure range of 0–320 kPa) at a loading speed of 50 mm/min. The sensing response of the developed pressure sensor remained substantially stable after 5000 loading–unloading cycles, indicating excellent repeatability and durability of the sensing unit. The dynamic resistance response in Figure 6a–e was constant with the results in Figure 5b. In addition, the developed sensing unit showed a fast response time (30 ms) and recovery time (90 ms) in the transient test, as illustrated in Figure 6f. To clearly quantify the repeatability, the data shown in Figure 6d were further processed and showcased in Figure 7. Figure 7a depicts that the non-repeatability (defined as the maximum deviation of three loading/unloading times divided by the entire output range in percentage) was about 5.9%, while that shown in Figure 7b was about 7.9%.

The above results illustrate that the flexible MWCNTs/PDMS pressure sensor had excellent and stable electromechanical characteristics, while it was manufactured by a simple fabrication process. All specific details about the developed sensors mentioned above were summarized in Table 2.

### 4.3. Integration of Flexible Insole Sensor

**Figure 8:**
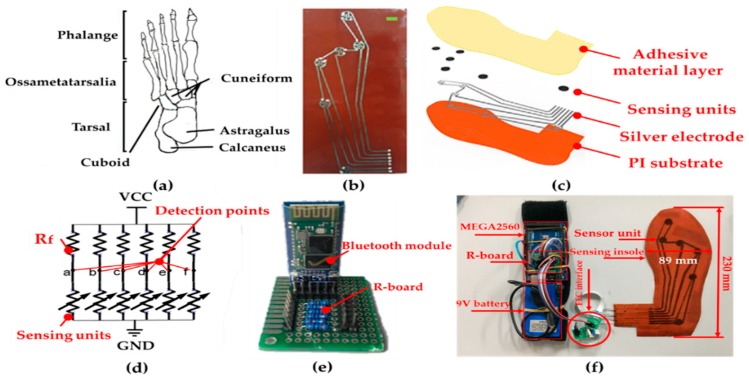
Integration of flexible pressure-sensitive insole. (**a**) Distribution of human foot bones. (**b**) The plantar inkjet-printed circuit diagram (scalar bar: 10 mm). (**c**) Distribution and assembly diagram of the sensing unit in the sole. (**d**) Principle of the voltage divider rule. (**e**) Voltage divider circuit and Bluetooth module on the R-board. (**f**) Plantar pressure detection system.

There are several key pressure points on a foot, which reflect gait information. If the pressure detection points are not selected properly, important gait information will be lost to some extent [42]. Figure 8a shows the natural bone composition of the human foot, which guides us to focus on some critical pressure detection areas on the plantar. Here, we measured six parts of plantar pressure distribution under dynamic and static conditions, including heel, arch, first metatarsal, second metatarsal, third to fifth metatarsal, and first toe. Therefore, we printed six interdigital electrodes in these six areas on PI substrate, as presented in Figure 8b. Then, we mounted six sensing units on the six printed interdigital electrodes, as shown in Figure 8c. As illustrated in Figure 8d, the readout circuit for resistive signals based on a voltage divider rule is widespread [43]. Here, we used this principle to detect resistances of six sensing units of the insole. In Figure 8e, in order to improve the comfort of wearing, we used wireless Bluetooth for signal transmission. As presented in Figure 8f, the micro control unit (MCU, MEGA2560), the circuit board (R-board), and the battery (9 V) were mounted on a wearable band. The R-board consisted of an array of resistive dividers. Then, we connected the flexible flat cable (FFC) with the smart pressure-sensitive insole by using silver conductive adhesive. Moreover, the MCU and the R-board were connected by conductive wires.

### 4.4. Gait Reference System Based on Vision

**Figure 9:**
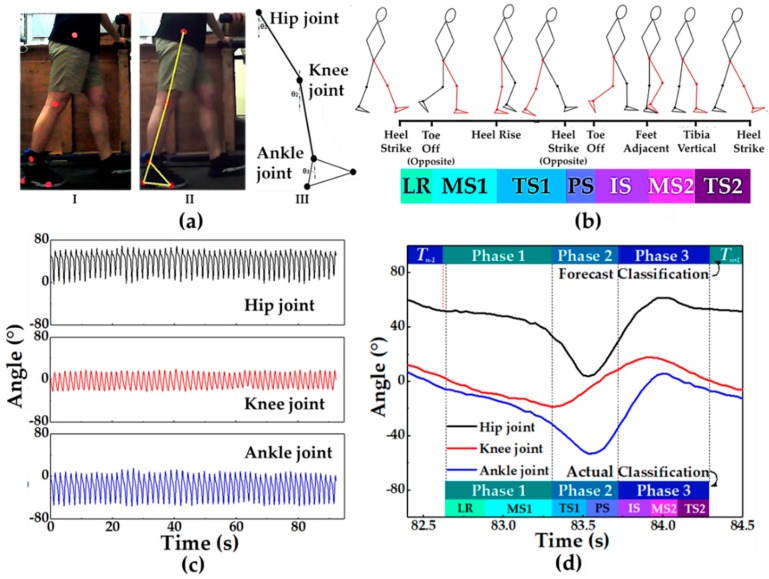
Gait reference system based on vision. (**a**) The process of extracting the joint angles of a leg: I. Distribution of leg markers. II. Skeleton model of the right leg. III. Extracted joint angles. (**b**) The basis of gait classification labels in machine learning [Perry’s method of dividing the period of gait, LR (loading response), MS1 (mid-stance), TS1 (terminal stance), PS (pre-swing), IS (initial swing), MS2 (mid-swing), TS2 (terminal swing). (**c**) Angle data of three joints (hip, knee, and ankle) extracted from the visual system after data processing. (**d**) The theoretical classification and the machine learning forecast classification of the phase of visual leg angles during a walking gait cycle.

The image processing method is one of the effective ways to divide the gait phase because it can obtain a great deal of information about gaits, such as leg swing angle, stride length, stride time, and stride frequency [44]. In order to facilitate comparison, reference, and analysis of gait signals collected by pressure-sensitive insole, we used the kNN machine learning method to recognize images captured by a camera (picture acquisition rate is 30 frames per second). The kNN machine learning model was trained to identify three gait phases by using three kinds of angles in each image. As shown in Figure 9a, we attached five markers to the hip, the knee, the ankle, the heel, and the toe of the volunteer’s right leg. Markers were identified by the MATLAB images recognition method. Then, the positions of the five markers were drawn on raw original pictures. After that, the shapes of the thigh, the calf, and the feet were sketched to show the gait of the volunteer, and the angle data of each joint were calculated based on the shape. After the above steps, we obtained images and angle data of volunteers walking (2 km per hour). Then, as shown in Figure 8c, we labeled the obtained angle data by referring to the corresponding photo according to the gait phase division method proposed by Perry [45], as shown in Figure 9b. Finally, by adjusting test-total ratio and K value, the recognition accuracy of gait recognition could be improved. After comparison (see more details in Section 5.2), it was found that when K = 1 and ratio = 0.1 (where ratio means prediction data as a proportion of the total data), the overall accuracy of the test was significantly improved. The success rate of recognition in almost all gait phases was up to 97%. The accuracy of Phase 2 and 3 was 100%, while that of Phase 1 was 93.5%. We applied the model to automatically classify the gait cycle in Figure 9d, and the results of the automatic phase classification of angles were almost consistent with those of manual phase classification. There was a sample point from Phase 3 of the former cycle that was incorrectly classified to Phase 1 of the next cycle.

### 4.5. Validation of Gait Phase Detection based on Flexible Insole Sensor

In order to demonstrate that the pressure-sensitive insole has a good static and dynamic response performance, a healthy adult male volunteer with a weight of 70 kg (volunteer #1, who also participated in the visual system experiment below) was invited to wear the pressure-sensitive insole. After obtaining the plantar pressure data of him, we analyzed the pressure distribution, classified his gait phase according to the classical gait classification criteria [45], and automatically forecasted classified gait phase through the kNN machine learning method.

**Figure 10:**
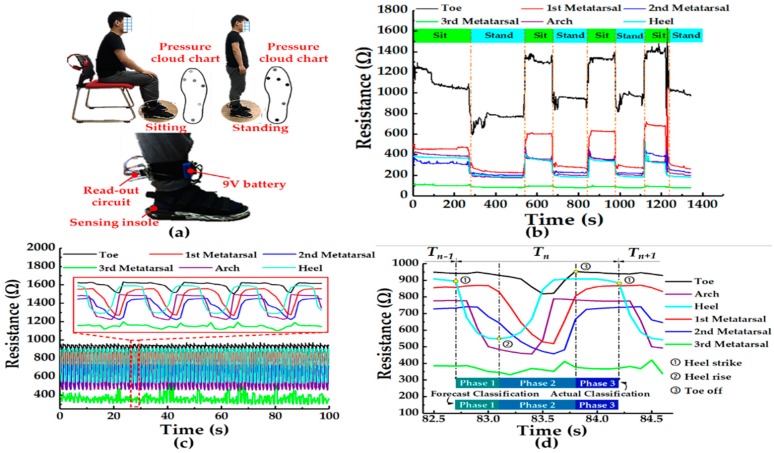
Pressure-sensitive insole function and wear demonstration. (**a**) Photos of the volunteer standing or sitting with the pressure-sensitive insole, the in-time pressure cloud diagrams of the six sensing units in the pressure-sensitive insole, and a zoom view of wearing status. (**b**) Resistance response of six sensing units in the pressure-sensitive insole during the volunteer standing and sitting. (**c**) Resistance response of six pressure-sensitive units on the insole while the volunteer was walking at 2 km/h. (**d**) The theoretical classification and the machine learning forecast classification of the phase of resistive pressure data during a walking gait cycle.

First of all, the pressure-sensitive insole can be comfortably worn on a foot. As shown in Figure 10a, after wearing the pressure-sensitive insole, the volunteer’s actions were not greatly hindered. To obtain the static response of the pressure-sensitive insole, the resistive data of the sensing units were recorded when the volunteer was sitting or standing. The pressure cloud chart of the plantar could be drawn according to the resistance changes of the sensing units. It was obvious that the color of the sensing points on the pressure cloud chart was darker when the volunteer was standing than that when he was sitting. When the volunteer was standing up, the body’s mass was all applied to the feet. Thereby, the pressure on the planar was higher than that in the sitting condition where the body’s mass was partly borne by the seated buttocks. After several standing–sitting cycles, we found that the changes in the resistance of each sensing unit had obvious repeatability in Figure 10b. This proved that the pressure-sensitive insole had good static performance. Walking is an appropriate way to test the dynamic characteristics of the pressure-sensitive insole. As shown in Figure 10c, 60-gait-cycle data were selected from the middle of total recorded cycles during the walking experiment. Therefore, the 60-gait-cycle data were regarded as steady state during the entire walking task. After analyzing the dynamic resistance-to-time curve of the pressure sensors, the results generally showed good reproducibility. Along with the repeat gait phase of the volunteer, the resistance also showed the phenomenon of reciprocating changes. In order to analyze the gait of the volunteers through resistance data and to automatically classify the data with machine learning later, here, we introduce Perry’s gait phase division method [45] (Figure 9b) as the basis for our gait division.

From a zoomed view of walking cycle resistance data in Figure 10d, the resistance variation trends of these sensing units were obviously different, because where the foot landed varied with gait phases. Approximately, a gait cycle of a single foot had a time interval of 1.7 s, which was equivalent to a stride frequency of 1.18 Hz. Since resistance data of the pressure-sensitive insole were collected only on the right foot, based on the above resistance data, the gait was divided into three phases during a walking cycle: Phase 1 (loading response and mid-stance), Phase 2 (terminal stance and pre-swing) and Phase 3 (initial swing, mid-swing, and terminal swing). A single walking cycle can be illustrated by the following description. The whole gait cycle starts from the landing of the heel of the right foot (marked as number 1 in Figure 10b), and the resistance of the sensing unit at the heel point decreases in the following short period. When the arch bone starts to be under pressure, the force on the heel starts to decrease, which means the heel is going to rise (marked as number 2 in Figure 10d). This is also known by the dropped resistance of the sensor unit located at the arch bone point and the rise in resistance of the sensor unit located at the heel point. Phase 1 (including loading response and mid-stance) is defined by the interval from marked number 1 to number 2 in Figure 10d. Then, this is followed by a dropped resistance of the sensing unit both located at the toe point and the metatarsal bone point, which means that the pressure of these areas is increasing. Meanwhile, overall plantar pressure is centralized on the forefoot. As the left foot lands, the left foot takes over from the right foot as the main supporting foot. Next, the resistance of the sensing unit located at the toe point and the metatarsal bone point increases due to the decreased pressure loaded on these areas. Finally, the resistance of the sensing unit located at the toe point, as the last unit, returns to a non-pressurized resistance value, which is symbolized as number 3 in Figure 10d to mark the end of Phase 2 (terminal stance and pre-swing). The dangling state of the right foot is classified as Phase 3 (initial swing, mid-swing, and terminal swing) because, during this phase, the resistances of sensing units are almost constant.

**Figure 11:**
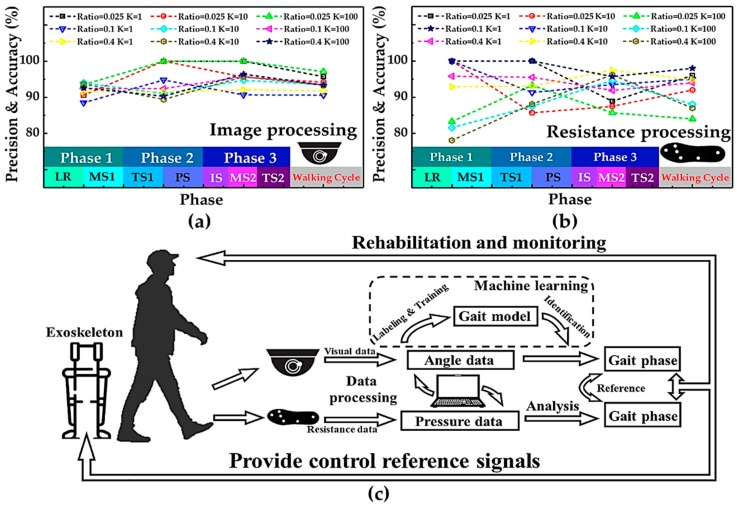
Comparison and combination of the pressure-sensitive insole and visual system. (**a**) The accuracy of visual data recognition in each phase of gait in machine learning varies with the change of parameters in the k-nearest neighbor (kNN) method. (**b**) The accuracy of resistive pressure data recognition in each phase of gait in machine learning varies with the change of parameters in the kNN method. (**c**) Schematic diagram of plantar pressure distribution and gait division analysis system with visual aid system.

Since the resistive data of walking cycles had good repeatability, we adopted the machine learning method to process the data and then automatically classified the gait phase. Here, we used the basic kNN method to classify the phase. After adjusting parameters and comparing accuracy (see more details in Section 5.2), we determined the set-up parameters (K = 100, ratio = 0.025). As shown in Figure 11b, the total recognition accuracy of visual-based gait information was up to 98%. The accuracy of Phase 1 and 2 was 100%, while that of Phase 3 was 96%. We applied the model to automatically classify the gait cycle in Figure 9d, and results (illustrated in Figure 10d) of automatic phase classification of resistive data were completely consistent with those of manual phase classification. These results reflected that the resistive data set collected by the pressure-sensitive insole was suitable for automatic classification by the machine learning kNN method.

## 5. Evaluation and Analysis

### 5.1. Key Factors of kNN Algorithm and Data Processing

The kNN algorithm, as a basic kind of non-parametric methods, is used for classification and regression [46]. The principle of the kNN algorithm is that the labeled data sets constitute a point cloud in the n-dimensional space, and the raw data are put into the n-dimensional space. The data to be identified are automatically classified into the label of which the number in a certain category is the largest among the K nearest existing data of the data to be identified. Appropriate K value and training ratio parameters in kNN algorithm can effectively improve the accuracy of automatic classification. We altered K values (1, 5, 10, 50, and 100) and training ratio parameters (0.025, 0.1, and 0.4) int he kNN algorithm in turn to make the model more suitable for the classification of visual data and resistance data.

With the continuous development of mobile Internet, most wearable systems have equipped with the smart phone to transport and process wearable data in a cost-effective manner [47]. However, the limited power and computational capabilities of a smart phone may have impacts on the complex tasks of data transportation and processing. To address these issues, wearable systems based on the Internet-of-Things (IoT) cloud was proposed, enhanced by powerful severs where the wearable data can be stored and analyzed efficiently for computation-intensive data processing [48]. The IoT-cloud technology is promoting the new revolution of wearable healthcare system, called Wearable 2.0, one primary challenge of which is the end-to-end latency limiting the application in real time monitoring [49]. To cope with this challenge, edge computing has attracted many researchers to devote significant efforts to developing a novel approach by leveraging additional processing resources [50]. Moreover, with the advanced technological innovations and massive production in hardware, on-device data analysis without the reliance on the cloud is possible [51]. In this work, all collected data were wirelessly transferred to a personal computer (PC) via Bluetooth for data storage and off-line data analysis. The communication tool for data storage between PC and flexible insole sensors was based on MATLAB. The post-processing of data classification based on the machine-learning method was also conducted on the same PC separately. In the future, we hope to deploy all algorithms on a compactable processer integrated with flexible insole sensors to explore the feasibility of on-device data analysis.

### 5.2. Performance Evaluation and Comparison of Classifiers

Appropriate K value and training ratio parameters in kNN algorithm can effectively improve the accuracy of automatic classification. We altered K values (1, 5, 10, 50, and 100) and training ratio parameters (0.025, 0.1, and 0.4) in the kNN algorithm in turn to make the model more suitable for the classification of visual data and resistance data. To completely evaluate the classifier, we computed the related assessment parameters of the training results, including total accuracy, precision, recall rate, and F1score, which are summarized in Table A1, Table A2, Table A3 and Table A4, respectively. These data can illustrate the effect of automatic classification. In some appropriate parameters, these classification indicators are relatively high. This shows that our framework can be applied to the collection and the analysis of human gait data.

As shown in Figure 11a,b, by comparing the prediction accuracy of these two data sets, if the parameters are appropriate (K = 100, ratio = 0.025 for pressure sensors; K = 1, ratio = 0.1 for vision systems), the prediction accuracy of both can reach above 95% (97% for pressure sensors and 98% for vision system). In addition, the classifier precision of three phases (Phase 1, Phase 2, and Phase 3) that were divided based on visual data was 93%, 100%, and 100%, respectively, while that based on pressure sensors was 100%, 100%, and 95%, respectively. Moreover, the classifier F1-scores of the three phases that were divided based on visual data were 0.967, 0.971, and 0.977, respectively, while those of pressure sensors were 1.000, 0.957, and 0.978, respectively. What is more, it was found that the quality of the data set obtained by using the pressure-sensitive insole was not inferior to that obtained by the image processing method in the machine learning algorithm using kNN.

### 5.3. Statistical Analysis and Comparison of Two Methods

**Figure 12:**
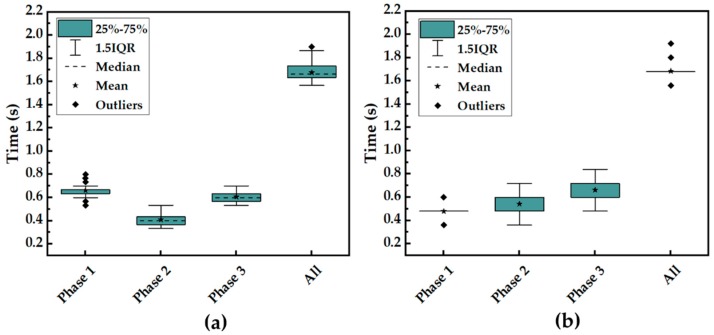
Comparison of stride time of different gait phases between vision systems and pressure insole sensors. (**a**) Statistical analysis of stride time based on vision systems. (**b**) Statistical analysis of stride time based on pressure sensors.

The statistical analysis of stride time based on visual signals and plantar pressure distribution was carried out, as shown in Figure 12. Although the total stride time of a walking cycle is almost the same, the time length of each gait phase divided by the two kinds of data was not consistent. This was attributed to the difference of labeling between visual data and pressure data. The visual data were classified based on the contact between the shoe and the treadmill, while plantar pressure data were classified based on the contact between the foot and the insole. There is a time difference between the two kinds of contact modes.

Meanwhile, vision and plantar pressure, as two kinds of information sources for gait phase recognition, have advantages over each other. For example, the heel strike and rise point can be recorded more accurately through the pressure-sensitive insole, while the feet adjacent and tibia vertical status could be obtained only through the visual system. Here, we combined the two gait recognition methods to make up for each other’s limitations, as shown in Figure 11c. The whole gait recognition system can provide a lot of visual and plantar pressure data for subsequent machine learning methods. By analyzing the characteristics between the two kinds of data, we hope to find the corresponding relationship between them. This method is beneficial for the monitoring of human health and the recovery of disease as well as for providing sufficient information for the control of an exoskeleton.

### 5.4. Adaptability Analysis of Pressure-Sensitive Insole

To verify that the results from the developed pressure-sensitive insole for gait phase division are generalizable, more volunteers were invited to participate in experiments. The detailed information about health condition of volunteers, including gender, weight, and body mass ndex (BMI (kg/m^2^), defined as a person’s weight in kilograms divided by the square of the person’s height in meters), are summarized in Table 3. All volunteers were requested to wear the developed pressure-sensitive insole while walking on a treadmill. The walking speed of treadmill was set as 2 km/h. The machine learning model used to process the data collected from these experiments was the same one with K = 100, ratio = 0.025. All the results, such as stride frequency, stride time, overall accuracy, precision, and F1-score, of five users are summarized in Table 4, illustrating the good adaptability of the pressure-sensitive insole.

## 6. Future Work

This paper focused on addressing the inherent design and implementation challenges with detailed prototype design and fabrication, an exploratory flexible sensors characterization experiment, and detailed experimental validation. In the current status, these pressure-sensitive insoles were not applied to the elderly, young children, disabled people, pregnant women, and other groups with special plantar pressure as well as a human exoskeleton to detect the heterogeneous distribution of plantar pressure. We look forward to further applying the developed devices to those areas until the compatibility of it is improved by the highly intensive integration design of all components, such as power supply, data acquisition, data processing, and data transmission. Meanwhile, our machine learning algorithm in the visual reference system is basic and can be adopted for classification. There is a need for developing a specific algorithm to tackle the challenges in accurate gait phase prediction considering possible effects of acceleration and deceleration phases at the start and the stop of the walking task. In future research, we plan to detect the plantar pressure distribution of more types of people under the whole walking process through this system, collect enough planar pressure and visual data, and try to establish individual differences in the corresponding relationship between plantar pressure and the angle of lower limb joints [52]. In this way, the exoskeleton control model can be trained to achieve the goal of personalized control in which exoskeleton control information is only obtained through plantar pressure signals.

## 7. Conclusions

In summary, a pressure-sensitive insole with the laser-processed surface was designed and fabricated. The highlight of the pressure sensors was that reduced and stabilized resistances of sensing units were obtained by the laser process. Furthermore, a pressure sensing insole was assembled by employing laser-processed sensing units and inkjet-printed electrodes. The characteristics of the pressure-sensitive insole were demonstrated by the experiment, where it was worn by a healthy human walking on a treadmill. The static characteristics of the pressure-sensitive insole were verified by the experiment of alternative standing and sitting. The dynamic characteristics of the pressure-sensitive insole were presented, the planar pressure signals obtained from the pressure-sensitive insole were analyzed by referring to the visual detection system based on machine learning, and the gait phase of the human was further divided. This work further compared the performance of the plantar pressure-sensitive insole with the image processing method by using machine learning. Finally, the gait monitoring system composed of the pressure-sensitive insole and visual aids is expected to provide an effective tool to obtain the corresponding data of lower extremity joint angle and plantar pressure to facilitate the subsequent exoskeleton control.

## Figures and Tables

**Table 1:** Comparison of the state-of-the-art design of wearable sensors for gait phase detection.

Sensor Type	Authors	Design Features	References
Optoelectronic sensors	Crea et al.	An insole with shading structures used optoelectronic signal to detect pressure	[38]
Inertial sensors	Ding et al.	The inertial measurement unit was attached onto the anterior surface of a shoe and measured the three-axis angular velocity and the acceleration of the foot	[10]
Electromyography (EMG) sensors	Joshi et al.	The reflective markers were attached on limbs to detect the EMG signal	[15]
Multi-axis force sensors	Lind et al.	Unique multi-axis foot force/torque sensors were integrated into a military style boot to measure the forces on the human foot	[39]
Conductive rubbers	Saito et al.	The flexible conductive rubber sensors were fixed to the insole with traditional circuit for electrical connection	[30]
	Our work	Conductive rubber sensors processed by laser cutting were fixed on flexible insoles.	

**Table 2:** Summary of characteristics of developed sensing unit.

Items	Parameters	Test Conditions
Detection range	0–25 N	Loading speed of 1 mm/min
Nonlinear errors	10.4%	Loading range of 0–5 N at a speed of 1 mm/min
	1.0%	Loading range of 5–15 N at a speed of 1 mm/min
	2.8%	Loading range of 15–25 N at a speed of 1 mm/min
Sensitivity	0.29 N^−1^ (3.63 MPa^−1^)	Loading range of 0–5 N at a speed of 1 mm/min
	0.12 N^−1^ (1.5 MPa^−1^)	Loading range of 5–15 N at a speed of 1 mm/min
	0.04 N^−1^ (0.5 MPa^−1^)	Loading range of 15–25 N at a speed of 1 mm/min
Repeatability	5.9%	Loading range of 0–25 N at a speed of 5 mm/min
	7.9%	Unloading range of 0–25 N at a speed of 5 mm/min
Hysteresis	8.9%	Cyclic force range of 0–25 N at a speed of 1 mm/min
Frequency response	0.012–1.25 Hz	Loading range of 0–20 N
Durability	5000 cycles	Loading range of 0–25 N at a speed of 50 mm/min
Response time	30 ms	Transient test (from 0 N to 2 N)
Recovery time	90 ms	Transient test (from 2 N to 0 N)

**Table 3:** Summary of detailed information of five volunteers.

Volunteer Number	Gender	Weight (kg)	Height (m)	BMI (kg/m^2^)
#1	Male	70	1.72	23.7
#2	Male	60	1.81	18.3
#3	Female	51	1.61	19.6
#4	Female	54	1.55	22.4
#5	Male	65	1.76	20.9

**Table 4:** Summary of results of gait phase division with several volunteers based on pressure-sensitive insole.

Volunteer Number	Stride Frequency (Hz)	Stride Time (s)	Overall Accuracy (%)	Precision (%)			F1-Score		
				Phase 1	Phase 2	Phase 3	Phase 1	Phase 2	Phase 3
#1	1.18	1.7	98.000	100.000	100.000	95.745	1.000	0.957	0.978
#2	1.12	1.8	96.000	100.000	100.000	91.667	1.000	0.933	0.857
#3	1.26	1.6	92.000	92.308	100.000	80.000	0.923	0.933	0.889
#4	1.34	1.5	88.000	92.308	60.000	100.00	0.923	0.750	0.875
#5	1.26	1.6	91.667	100.000	100.000	87.500	1.000	0.667	0.933

## Appendix A

**Table A1:** Comparison of overall accuracy based on volunteer #1 (unit: %).

Methods		K = 1	K = 5	K = 10	K = 50	K = 100
Vision systems	Ratio = 0.025	91.304	92.750	94.203	95.652	97.101
	Ratio = 0.1	90.614	90.614	93.502	93.863	93.501
	Ratio = 0.4	91.772	92.948	93.309	93.580	93.400
Pressure sensors	Ratio = 0.025	96.774	96.774	93.548	90.323	83.871
	Ratio = 0.1	98.000	95.000	95.000	92.000	88.000
	Ratio = 0.4	94.000	96.250	94.750	91.500	87.000

**Table A2:** Comparison of recall based on volunteer #1 (unit: %).

Methods		Phase	K = 1	K = 5	K = 10	K = 50	K = 100
Vision systems	Ratio = 0.025	1	100.000	100.000	100.000	100.000	100.000
		2	77.778	83.333	83.333	88.889	100.000
		3	90.909	90.910	95.455	95.455	100.000
	Ratio = 0.1	1	95.575	93.805	96.460	97.345	96.460
		2	79.710	84.058	88.406	89.855	88.406
		3	92.632	91.579	93.684	92.632	93.684
	Ratio = 0.4	1	92.706	93.412	94.353	93.882	94.824
		2	88.104	91.450	94.052	94.052	92.937
		3	93.024	93.447	91.748	92.961	92.233
Pressure sensors	Ratio = 0.025	1	100.000	100.000	100.000	100.000	100.000
		2	88.889	88.889	88.888	77.778	77.777
		3	100.000	100.000	90.909	90.909	72.727
	Ratio = 0.1	1	100.000	100.000	100.000	100.000	100.000
		2	91.667	83.333	88.888	77.778	77.777
		3	100.000	97.778	95.556	91.111	80.000
	Ratio = 0.4	1	95.763	98.305	100.000	100.000	100.000
		2	89.744	93.162	92.308	88.034	88.889
		3	95.758	96.970	92.727	87.879	76.364

**Table A3:** Comparison of precision based on volunteer #1 (unit: %).

Methods		Phase	K = 1	K = 5	K = 10	K = 50	K = 100
Vision systems	Ratio = 0.025	1	87.879	90.630	90.625	90.625	93.548
		2	100.000	100.000	100.000	100.000	100.000
		3	90.909	90.910	95.455	100.000	100.000
	Ratio = 0.1	1	88.525	92.174	93.s996	92.437	92.373
		2	94.828	89.231	91.045	92.537	92.424
		3	90.722	89.691	94.681	96.703	95.699
	Ratio = 0.4	1	91.628	94.076	93.692	93.662	92.644
		2	91.506	89.781	89.399	90.681	90.253
		3	92.086	93.092	95.696	95.511	96.447
Pressure sensors	Ratio = 0.025	1	100.000	100.000	100.000	91.667	78.571
		2	100.000	100.000	88.888	87.500	87.500
		3	91.667	91.667	90.909	90.909	88.888
	Ratio = 0.1	1	100.000	100.000	100.000	91.176	81.579
		2	100.000	95.238	91.304	90.909	87.500
		3	95.745	91.667	93.478	93.182	94.737
	Ratio = 0.4	1	95.763	96.667	92.913	84.286	78.146
		2	95.455	97.321	93.103	95.370	88.136
		3	91.860	95.238	97.452	95.395	96.183

**Table A4:** Comparison of F1-score based on volunteer #1.

Methods		Phase	K = 1	K = 5	K = 10	K = 50	K = 100
Vision systems	Ratio = 0.025	1	0.935	0.951	0.951	0.951	0.967
		2	0.875	0.909	0.909	0.941	0.971
		3	0.909	0.909	0.955	0.977	0.977
	Ratio = 0.1	1	0.919	0.930	0.952	0.948	0.944
		2	0.866	0.866	0.897	0.912	0.904
		3	0.917	0.906	0.942	0.946	0.947
	Ratio = 0.4	1	0.922	0.937	0.940	0.938	0.937
		2	0.898	0.906	0.917	0.923	0.916
		3	0.926	0.937	0.937	0.942	0.943
Pressure sensors	Ratio = 0.025	1	1.000	1.000	1.000	0.957	0.880
		2	0.941	0.941	0.889	0.824	0.824
		3	0.957	0.957	0.909	0.909	0.800
	Ratio = 0.1	1	1.000	1.000	1.000	0.954	0.899
		2	0.957	0.889	0.894	0.870	0.899
		3	0.978	0.946	0.945	0.921	0.867
	Ratio = 0.4	1	0.958	0.975	0.963	0.915	0.877
		2	0.925	0.952	0.927	0.916	0.885
		3	0.938	0.961	0.950	0.915	0.851
